# The Extirpation of Aneurysms

**Published:** 1899-03-04

**Authors:** 


### THE EXTIRPATION OF ANEURYSMS.

At the meeting of the Royal Medical and Chirurgical Society last Tuesday a case was related by Mr. Walsham, in which he had treated a large aneurysm of the carotid by extirpation. The patient, a strong, healthy-looking man, aged 49 years had noticed a small swelling on the right side of his neck for six years past. It had increased slowly and without pain until a few months before he came under observation, since which time it had got rapidly larger. The swelling extended from the angle of the jaw nearly to the clavicle, and from near the middle line in front passed deeply beneath the sterno-mastoid muscle. It was generally hard in consistence, but was soft and fluctuating in places. The skin was not adherent, and the swelling had no connection with the larynx or bones. There was neither pulsation nor bruit, and nothing escaped on puncture with an exploring syringe. The case was discussed at the ordinary weekly consultation, and the swelling was thought to be glandular and probably malignant. On an exploratory incision being made, however, the tumour was discovered to be an aneurysm, but even at the time of the operation no pulsation could be felt. The common carotid was ligatured in two places below the sac and divided between the ligatures, the sac was dissected up, collateral vessels were secured as they were met with, and the internal carotid, which was found to be considerably dilated, was ligatured above. The external carotid was not recognised, but was probably one of the numerous vessels tied. The sac of the aneurysm was removed entire, and the patient made a good recovery. One point of interest in the case was the absence of pulsation, but it was suggested by Mr. Walsham that perhaps it might have been found to exist on its inner side towards the pharynx if examination had been made in that direction. The cause of non-pulsation in the few recorded cases of this nature is not very evident, but is generally attributed either to leaking of the sac or to blocking of its mouth with clots; it was suggested, however, in the discussion that it might possibly be caused by a part of the aneurysm pressing on the artery below. It was also pointed out during the discussion that although recorded cases of aneurysm without pulsation are rare, there is probably a time in the history of every aneurysm which undergoes spontaneous cure when it is without pulsation. In considering the various modes of treatment which are available for the cure of aneurysm, Mr. Walsham pointed out that the following are the conditions which appear to be most favourable for extirpation: (1) Where there is insufficient room to apply a ligature on the proximal side, or where a proximal ligature is attended with great risk, as ligature of the innominate for subclavian aneurysms. (2) Where a number of large vessels communicate with the sac. (3) Where other measures have failed to cure the aneurysm. (4) Where the aneurysm, as in the popliteal artery, has become diffused, or rupture of the sac or gangrene of the limb is threatened. (5) Where the setting free of emboli, as in carotid aneurysms, would be attended with risk of cerebral softening. But the author of the paper carefully guarded himself from being thought to suggest that the operation of enucleation should be looked upon as the best in all cases, and he especially insisted upon the difficulties which might occur in its application to popliteal aneurysms in which it could hardly compare with such a simple and, as a rule, satisfactory procedure as ligature of the popliteal or even of the femoral at the apex of Scarpa's triangle. In this view he was thoroughly supported by most of the speakers in the discussion.

